# Multidisciplinary Timely Debrief Sessions after Cardiac Arrests and Endotracheal Intubations in the Pediatric Emergency Department

**DOI:** 10.1097/pq9.0000000000000919

**Published:** 2026-09-28

**Authors:** Noreen Bux

**Affiliations:** From the Department of Medicine, Liaquat University of Medical and Health Sciences, Jamshoro, Pakistan.

We read with great interest the quality improvement study titled “Multidisciplinary Timely Debrief Sessions after Cardiac Arrests and Endotracheal Intubations in the Pediatric Emergency Department,”^1^ which describes the implementation of a standardized multidisciplinary debriefing protocol following pediatric emergency department cardiac arrests and endotracheal intubations. The authors deserve credit for tackling an important but understudied dimension of emergency care and for showing that a structured, technology-based debriefing process can substantially improve debrief completion without compromising patient care. Beyond improving debrief completion, the multidisciplinary approach reinforces collaborative practice and promotes a culture of reflection and continuous quality improvement within the pediatric emergency department. The intervention was successful in significantly improving the rate of debrief completion, but the primary outcome did not include patient-centered outcomes, only process measures.

Consequently, it is uncertain whether greater debrief utilization resulted in enhancements in the quality of cardiopulmonary resuscitation, patient survival, neurologic outcomes, or decreases in preventable adverse events. Moreover, debrief completion was defined as the submission of a REDCap form and did not assess debrief fidelity (eg, adherence to the intended framework, multidisciplinary participation, or quality of facilitated discussion). Future evaluations could be strengthened by including validated debrief assessment tools, as evidence suggests that high-quality debriefing, rather than merely conducting a debrief, influences learning and team performance.^2^ Finally, the authors correctly highlight the psychological and educational benefits of debriefing; however, the well-being, psychological safety, and burnout of healthcare providers were not measured with validated instruments.^3^ Further multicenter studies that incorporate patient-centered clinical outcomes, standardized measures of debrief quality, and validated measures of healthcare worker well-being would provide more comprehensive evidence regarding the effectiveness and broader impact of structured postevent debriefing. Future studies should measure the completion of debriefing as well as the quality and fidelity of debriefing using validated assessment instruments. Implementation research exploring the sustainability of debriefing practices after the withdrawal of intensive educational support and local quality improvement champions would also be informative about long-term integration into routine clinical workflows. Due to the multimodal nature of the intervention, it should identify the relative contribution of individual implementation strategies, such as structured facilitation, accessibility of QR codes, and targeted education, to identify the most efficient and scalable approach. Finally, formal cost-effectiveness analyses would help to determine if broad dissemination of structured multidisciplinary debriefing is a sustainable investment for diverse healthcare settings.
